# Indigenous peoples and pandemics

**DOI:** 10.1177/14034948221087095

**Published:** 2022-05-12

**Authors:** Daniele E. Alves, Svenn-Erik Mamelund, Jessica Dimka, Lone Simonsen, Mathias Mølbak, Søren Ørskov, Lisa Sattenspiel, Lianne Tripp, Andrew Noymer, Gerardo Chowell-Puente, Sushma Dahal, Taylor P. Van Doren, Amanda Wissler, Courtney Heffernan, Kirsty Renfree Short, Heather Battles, Michael G. Baker

**Affiliations:** 1 Centre for Research on Pandemics & Society, Oslo Metropolitan University, Norway; 2 PandemiX Center, Roskilde University, Denmark; 3 Department of Anthropology, University of Missouri, USA; 4 Department of Anthropology, University of Northern British Columbia, Canada; 5 Program in Public Health, University of California, Irvine, USA; 6 Department of Population Health Sciences, Georgia State University, USA; 7 School of Human Evolution and Social Change, Arizona State University, USA; 8 Tuberculosis Program Evaluation and Research Unit, University of Alberta, Canada; 9 School of Chemistry and Molecular Biosciences, University of Queensland, Australia; 10 Department of Anthropology, University of Auckland, New Zealand; 11 Health Environment Infection Research Unit, University of Otago, New Zealand

**Keywords:** 1918 influenza pandemic, 2009 influenza pandemic, COVID-19, pandemic preparedness, mortality, Indigenous peoples, social inequalities, social determinants of health infectious diseases

In addition to posing a major threat to global health, pandemics impact economic activity, as witnessed during the spread of COVID-19 around the globe. The disease risks, however, are not uniform for major pandemic threats. For example, risk groups for severe disease during seasonal epidemic influenza, the influenza pandemics of 1918 and 2009, and the ongoing COVID-19 pandemic are different. The 1918 and 2009 influenza pandemics largely killed young adults, while the COVID-19 pandemic has primarily killed the elderly. Indeed, age is the strongest risk factor for severe outcomes of COVID-19. Within age groups, however, persons with underlying medical risk factors, people of lower socioeconomic status, immigrants, ethnic minorities, and Indigenous peoples are at higher risk of infection, hospitalization, and death across these pandemics and epidemics, demonstrating a need for intersectional analyses and preparedness responses [1].

A recent study has shed light on the substantial excess mortality due to COVID-19 that has been seen in many countries [2]. Despite this and other extensive epidemiological investigations, data and research on the global effect of COVID-19 on Indigenous groups remains strikingly scarce. One review from 2021 on global patterns of data collection among Indigenous peoples found that only nine out of 195 countries reported on mortality due to COVID-19 by Indigenous identity [3]. Another review concluded that this lack of data and research supports only a low-confidence conclusion on mortality while there was insufficient evidence to draw conclusions for other disease outcomes [4].

We performed a summary of the data in one of these reviews [3] and other studies [5–11] to test the hypothesis that Indigenous populations, globally, are more likely to experience mortality from COVID-19 than non-Indigenous populations. We found studies from nine countries (Australia, Brazil, Canada, Ecuador, Mexico, Peru, Colombia, New Zealand, and USA). If data allowed it, we calculated three pandemic outcomes by Indigenous and non-Indigenous status and calculated the Indigenous to non-Indigenous rate ratios for those outcomes. Those three outcomes were:

1. AR = attack ratio (underestimated, probably especially so in marginalized populations) is defined as:

$$AR=\frac{Confirmed\;COVID19\;cases}{Population\;size}$$

2. CFR = case fatality ratio (overestimated, probably especially so in marginalized populations) is defined as:

$$CFR=\frac{Confirmed\;COVID19\;\;deaths}{Confirmed\;COVID19\;cases}$$

3. PFR = population fatality ratio (based on official deaths, probably also underestimated, especially in marginalized populations, but less so than AR – it is easier to count deaths than cases) is defined as:

$$PFR=\frac{Confirmed\;COVID19\;deaths}{Population\;size}$$

The rate ratios were calculated as:

$$\begin{array}{l} ARR=\frac{AR_{Indigenous}}{AR_{non-Indigenous}},\\ \;\;CFRR=\frac{CFR_{Indigenous}}{CFR_{non-Indigenous}},\\ \;\;PFRR=\frac{PFR_{Indigenous}}{PFR_{non-Indigenous}} \end{array}$$

Of the results from the nine countries, six (Australia, Brazil, Canada, Ecuador, Mexico, Peru) have reported lower COVID-19 mortality in Indigenous populations. In Australia, however, COVID-19 cases in Aboriginal communities were too low to fully understand SARS-CoV-2-mediated morbidity in mortality. Peru and Brazil observed an overall decreased mortality burden in Indigenous populations, but it was increased in the Ucayali region in Peru and Amazon region in Brazil. The mortality burden in Indigenous populations was higher than for the non-Indigenous in three countries (Colombia, New Zealand, and USA).

As shown in Table I, most of the results refute our hypothesis of higher COVID-19 mortality among the Indigenous population compared with the non-Indigenous population. Instead, the results indicate that with respect to in-country mortality, Indigenous peoples fared better than non-Indigenous populations. Given a profound risk of bias and true demographic differences these crude comparisons should be interpreted with caution. For example, concerns of under-reporting of COVID-19 disease status attributable to inaccessible testing by ethnicity and geography (i.e. in remote areas) could severely bias these findings. If Indigenous groups are tested less than non-Indigenous, say, the CFRs would be erroneously higher in Indigenous populations. Or, if COVID-19 deaths are less well ascertained in Indigenous groups, then the risk estimates would be erroneously too low in comparison non-Indigenous groups. In some of these studies the CFR is very high in both groups, which is evidence of limited testing. Finally, several countries, for example, Norway, do not report cases, hospitalizations, or death for their Indigenous populations. The Norwegian epicenter of the COVID-19 pandemic has been Oslo, located in the southeastern part of the country, while most of the Indigenous Sámi people live in Northern Norway. Considering the lower number of cases in Northern Norway, and the fact that the living conditions, access to health services, and education are much more similar to the majority society than is the case for other Indigenous peoples, globally, it is less likely that there are Indigenous versus non-Indigenous differences in the COVID-19 disease burden in Norway (but such differences were indeed present 100 years ago during the 1918–1920 influenza pandemic; see below).

**Table I.** Is there evidence in the academic literature for our hypothesis of a higher COVID-19 pandemic mortality in Indigenous than in non-Indigenous populations?.

Reference	Country/region	Month of publication	Non-Indigenous (NI) AR, CFR, PFR estimates	Indigenous (I) AR, CFR, PFR estimates	Ratios (I/NI) ARR, CFRR, PFRR	Strength of evidence
	**North America**					
5	Canada	May 2020	No data available.	No data available.	No data available.	No data available.
3^a^	Canada	March 2021	AR: 0.87% CFR: 10% PFR: 0.090%	AR: 0.27% CFR: 1% PFR: 0.0029%	ARR: 0.31 CFRR: 0.10 PFRR: 0.033	Strong evidence against hypothesis.
3^a^	USA, Navajo Nation	March 2021	AR: 3.8% CFR: 2% PFR: 0.078%	AR: 4.2% CFR: 4% PFR: 0.18%	ARR: 1.1 CFRR: 2.0 PFRR: 2.3	Strong evidence in favor of hypothesis.
3^a^	USA, Alaska Natives, 23 states	March 2021	AR: 0.17%	AR: 0.59%	ARR: 3.5	No data on mortality.
3^a^	USA, Alaska Natives, 1 May–31 August 2020	March 2021	PFR: 0.034%	PFR: 0.064%	PFRR: 3.51	Lacking data, but in favor of hypothesis.
6	USA	August 2020	PFR: 0.032%	PFR: 0.061%	PFRR: 1.9	Lacking data, but in favor of hypothesis.
7	USA, 14 states	December 2020	PFR: 0.030%	PFR: 0.56%	PFRR: 1.8	Lacking data, but in favor of hypothesis.
	**South America**					
3^a^	Brazil, total population	March 2021	AR: 2.9% CFR: 3% PFR: 0.080%	AR: 4.4% CFR: 3% PFR: 0.077%	ARR: 1.5 CFRR: 0.79 PFRR: 0.96	Strong evidence of no difference.
8	Brazil, the Amazon	April 2021	AR: 2.335% CFR: 2.6% PFR: 0.0695%	AR: 5.524% CFR: 3.0% PFR: 0.146%	ARR: 2.37 CFRR: 1.15 PFRR: 2.10	Strong evidence in favor of hypothesis.
3^a^	Colombia	March 2021	AR: 2.4% CFR: 2% PFR: 0.045%	AR: 2.3% CFR: 4% PFR: 0.083%	ARR: 0.95 CFRR: 2.0 PFRR: 1.8	Strong evidence for hypothesis: double risk
3^a^	Ecuador	March 2021	AR: 1.1% CFR: 7% PFR: 0.077%	AR: 0.070% CFR: 3% PFR: 0.0023%	ARR: 0.066 CFRR: 0.45 PFRR: 0.029	Weak evidence against hypothesis.
3^a^	Mexico	March 2021	AR: 0.83% CFR: 10% PFR: 0.080%	AR: 0.091% CFR: 11% PFR: 0.0098%	ARR: 0.11 CFRR: 1.1 PFRR: 0.12	Weak evidence; high degree of case under-ascertainment.
9	Mexico	May 2020	No data available.	No data available.	No data available.	No data available.
10	Mexico	April 2021	CFR: 11% (44,986/407,548)	CFR=17% (768/4469)	CFRR=1.6	Very high under-ascertainment. Inconclusive data.
3^a^	Peru, total population	March 2021	AR: 3.1% CFR: 4% PFR: 0.12%	AR: 13% CFR: 1% PFR: 0.074%	ARR: 4.0 CFRR: 0.15 PFRR: 0.62	Strong evidence against hypothesis.
3^a^	Peru, Ucayali region	March 2021	AR: 3.5% CFR: 2% PFR: 0.071%	AR: 4.7% CFR: 7% PFR: 0.30%^b^	ARR: 1.33 CFRR: 3.25 PFRR: 4.31^b^	Weak evidence due to different death reporting methods.
	**Oceania**					
3^a^	Australia	March 2021	AR: 0.11% CFR: 3% PFR: 0.035%	AR: 0.018% CFR: 0% PFR: 0%	ARR: 0.17 CFRR: 0 PFRR: 0	Weak evidence against hypothesis, few cases.
3^a^	New Zealand	March 2021	AR: 0.036% CFR: 1% PFR: 0.050%	AR: 0.022% CFR: 3% PFR: 0.059%	ARR: 0.60 CFRR: 2.0 PFRR: 1.2	Weak evidence – small numbers.
11	New Zealand	September 2020	No data available.	No data available.	No data available.	Modelling study, not based on data – unfounded assumptions. No data.

a Reference number 3 is a review containing many other references to data used in their article and the table above.

b Based on self-reported deaths by Indigenous leaders in the Ucayali region up to 30 July 2020.

AR: attack ratio; CFR: case fatality ratio; PFR: population fatality ratio; ARR: AR rate ratio; CFRR: CFR rate ratio; PFRR: PFR rate ratio.

We also note that none of the studies seeks to adjust for marked demographic or socioeconomic differences in Indigenous and non-Indigenous populations [3,5–11]. The most important demographic differences are lower mean age and fewer elderly among Indigenous peoples than among non-Indigenous populations; both are factors that would tend to lead to lower unadjusted mortality in the entire population. In other words, a study observing no difference in CFR between American Indians and White Americans could be biased by the underlying demography. In the US, the median age of the White population is 43.7 years and the proportion >65 years is 16.5%, while for Native Americans, the median age is 31.2 years and the proportion >65 years is 7.4%. As the most important risk factor for COVID-19 mortality is age, a true difference in case fatality rates could thus be masked by differences in mean age and proportion of elderly. Even a 1:1 ratio of deaths could thus be a sign of increased vulnerability in Indigenous populations since they are younger globally.

We agree with the conclusion of a prior review [3], that there are simply not enough high quality data, which makes it difficult to investigate whether Indigenous peoples have a larger COVID-19 mortality risk than non-Indigenous persons. For future investigations, Indigenous and non-Indigenous researchers should 1) collaborate with Indigenous communities and stakeholders; 2) ARs using representative serology/antibody data and infection fatality ratio should be used rather than ARs based on lab-confirmed cases and the corresponding CFR; and 3) at a minimum, researchers should control for age but preferably also other medical and social risk factors.

Despite the limited research on the current COVID-19 pandemic with respect to Indigenous groups, historical analyses have shown disproportionate infectious disease outcomes in Indigenous groups. Indigenous populations provide examples of ethnic groups who are uniquely at risk for severe disease and death due to pandemic and seasonal influenza both today and 100 years ago [12–21].

For example, during the 1918–1920 pandemic, the Indigenous Māori in New Zealand had 4–6 times higher mortality risk than non-Indigenous people; the Sámi population in Norway had seven times higher mortality risk than non-Indigenous Norwegians[22,23]. Some very isolated Inuit villages in Alaska and Labrador, which presumably had little to no childhood experiences with pandemic, epidemic or endemic influenza, were worst affected by the new virus. In Brevig, Alaska, 90% of the inhabitants died, and Okak, Labrador was abandoned after suffering a mortality rate of 78% [15].

During the 2009 pandemic, Indigenous peoples in North America, Oceania, and the Pacific had 3–8 times higher pandemic mortality than the majority populations [17,19]. This disparity may be explained in part by a higher prevalence (2–7 times higher) of risk factors for severe influenza outcomes among the Indigenous (e.g. diabetes mellitus, obesity, chronic obstructive pulmonary disease, and a greater number of pregnancies at young age). Less documented factors include those associated with an increased risk of infection (e.g. crowding, family size, and poverty), unequal access to health care, lower health literacy and lower consumption of health services, and less genetic variability [13]. Furthermore, Indigenous populations historically have been geographically isolated, causing their lifetime exposure to influenza and immunological profiles to be different from non-Indigenous groups’. Isolation may also be a manufactured vulnerability as many Indigenous populations have been dispossessed from their homelands during times of colonization. Finally, although Indigenous populations in the USA, Canada, and Australia [24,25] are prioritized for both seasonal and pandemic influenza vaccines, lower vaccination rates among Indigenous groups than among non-Indigenous populations may also explain the disparities [13,26,27].

The reasons for the disproportionately poor health outcomes among Indigenous populations are complex, poorly understood, and represent an area of limited research, not only because of data issues, but also because existing epidemiological, genetic, and social science research is typically carried out in isolation, without engaging in interdisciplinary conversations. More importantly, this research may be done without the direct involvement of the affected Indigenous groups. Research on influenza in Indigenous peoples, as well as on preparedness planning that emphasizes Indigeneity as a risk factor, often focuses on a single region at a time, predominantly in either North America [28] or Oceania [29], adding to the fragmented understanding. Aside from this fragmentation, these studies are further limited because Indigeneity is not in itself a risk factor, nor is it a homogeneous identity. Combining biological and social science perspectives will help scholars, politicians, and other stakeholders to understand why Indigenous peoples in historical and modern times suffer from higher rates of infection, hospitalization, and mortality during prior influenza pandemics. Some studies have examined the role of key proteins for effective and broad cross-reactive killer T cell immunity Human leukocyte antigens (HLAs) in Indigenous peoples worldwide for severe influenza pandemic outcomes [30,31], but to date this research considers only genetic variability. Most social scientists and historians focus primarily on social and contextual factors (e.g. colonization), and do not take biological and environmental differences into account.

## Interdisciplinary research and pandemic preparedness needed

Ongoing and future pandemics may revive painful intergenerational trauma in Indigenous populations that may exacerbate their disease burden. Historically, many Indigenous groups in the Americas were decimated by colonial diseases such as smallpox or measles [32,33]. Potential vulnerability and disparate outcomes cannot be isolated or reduced to genetics or Indigenous culture without considering their historical and current regional and national context, including the degree of national preparedness, uptake of (non-)pharmaceutical interventions, extent of collaborative responses between governments and Indigenous populations, and the development and availability of culturally sensitive and affordable health services. In other words, we need interdisciplinary research and pandemic planning on topics incorporating Indigenous scholars’ and communities’ experiences, perspectives, and priorities. Such interdisciplinary and trans-continental efforts cannot exclude lessons learned from earlier pandemics – especially those on the interactions of contextual and social/medical risk factors – to mitigate the disease burden and consequences of the current pandemic and to prepare for future pandemics.
